# Parental perception of built environment characteristics and built environment use among Latino families: a cross-sectional study

**DOI:** 10.1186/s12889-016-3854-7

**Published:** 2016-11-22

**Authors:** William J. Heerman, Stephanie J. Mitchell, Jessica Thompson, Nina C. Martin, Evan C. Sommer, Margaret van Bakergem, Julie Lounds Taylor, Maciej S. Buchowski, Shari L. Barkin

**Affiliations:** 1 Department of Pediatrics, Vanderbilt University Medical Center, 2146 Belcourt Ave, 2nd Floor, Nashville, TN 37209 USA; 2 Independent Research Consultant, Nashville, TN USA; 3 Department of Psychology and Human Development, Peabody College, Vanderbilt University, Nashville, USA; 4 Center for Geospatial Analytics, North Carolina State University, Raleigh, NC USA; 5 Department of Medicine, Energy Balance Laboratory, Division of Gastroenterology, Hepatology and Nutrition, Vanderbilt University, Nashville, USA; 6 Vanderbilt Kennedy Center, Vanderbilt University, Nashville, USA

**Keywords:** Obesity, Physical activity, Built environment

## Abstract

**Background:**

Perception of undesirable features may inhibit built environment use for physical activity among underserved families with children at risk for obesity.

**Methods:**

To examine the association of perceived availability, condition, and safety of the built environment with its self-reported use for physical activity, we conducted a cross-sectional analysis on baseline data from a randomized controlled trial. Adjusted Poisson regression was used to test the association between the primary independent variables (perceived availability, physical condition, and safety) with the primary outcome of self-reported use of built environment structures.

**Results:**

Among 610 parents (90% Latino) of preschool-age children, 158 (26%) reported that there were no available built environment structures for physical activity in the neighborhood. The use of built environment structures was associated with the perceived number of available structures (B = 0.34, 95% CI 0.31, 0.37, *p* < 0.001) and their perceived condition (B = 0.19, 95% CI 0.12, 0.27, *p* = 0.001), but not with perceived safety (B = 0.00, 95% CI −0.01, 0.01, *p* = 0.7).

**Conclusions:**

In this sample of underserved families, perceived availability and condition of built environment structures were associated with use rather than perceived safety. To encourage physical activity among underserved families, communities need to invest in the condition and availability of built environment structures.

**Trial registration:**

Registered at ClinicalTrials.gov (NCT01316653) on March 11, 2011.

## Background

In the United States, the epidemic of childhood obesity disparately affects traditionally underrepresented minority groups, specifically Latinos and African Americans [1]. Regular participation in physical activity is part of a healthy lifestyle that can improve obesity-related health outcomes [2]. However, many children, especially those from traditionally under-represented minority groups, do not meet recommended daily amounts of physical activity [3, 4]. Two factors that are critical to young children’s participation in physical activity are parenting practices (e.g., engagement in child’s activity) and access to outdoor play space, where preschool age children’s highest intensity physical activity typically occurs [5, 6]. Thus, both parental perception and use of the built environment may be important factors related to the childhood obesity epidemic.

Features of the built environment -“the human-made space in which people live, work, and recreate on a day-to-day basis” [7] - may be modifiable barriers to achieving recommended physical activity goals [8]. Use of the built environment for physical activity is dependent on personal and social factors as well as at least three intrinsic features of built environment structures: availability, condition, and personal safety [9]. Previous work has shown that a parent’s perception of these three intrinsic built environment features is more strongly associated with parenting behaviors that support child physical activity than objective measures of built environment safety [10]. Consequently, studying the effects of parent perception of the intrinsic features of their built environment is an important approach to identifying modifiable determinants of childhood physical activity.

Because a parent’s negative perception of a neighborhood’s physical environment has been previously associated with parenting practices that discourage child use of the built environment, the purpose of the current study was to examine the relationships between parent-perceived availability, condition, and safety of the built environment and parental use of built environment structures for physical activity, controlling for both individual and social factors. This analysis advances the field in two ways. First, we utilized a newly developed composite measure of safety of the built environment including salient features for parents of young children (i.e., crime safety, traffic safety, walkability, bikeability) [11]. Second, we considered built environment and physical activity within an underserved minority population who experience disproportionate rates of childhood obesity and crime, and thus our findings can inform community/environmental policies and programming tailored to this at-risk population. We hypothesized that a greater number of available built environment structures as well as parents’ perception of both better condition and greater safety of the built environment would be associated with higher use of built environment structures for physical activity.

## Methods

Data used in the current analyses were collected at baseline prior to randomization in an on-going randomized controlled trial of a parent-child intervention designed to prevent childhood obesity [12]. Study procedures were approved by the Institutional Review Board of Vanderbilt University Medical Center. The trial is registered at ClinicalTrials.gov (NCT01316653).

### Participants

**Fig. 1:**
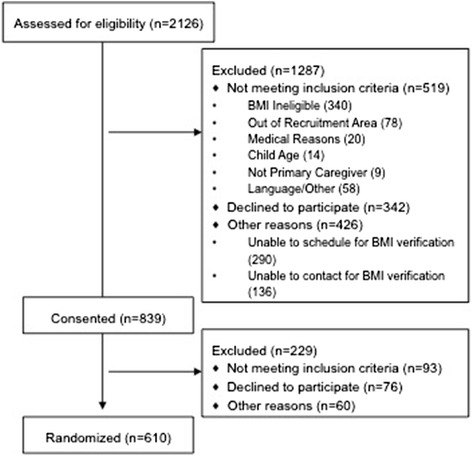
CONSORT Flow Diagram for Study Enrollment

Parent-preschool child dyads were recruited from Davidson County, Tennessee. To ensure similar geographic accessibility to built environment structures, participants resided in or self-identified as users (at least once per week) of the built environment in one of two zip code regions contiguous with two collaborating community recreation centers. Dyads were eligible to participate if they received at least one form of government assistance, spoke English or Spanish, the parent was over 18 years old, the child was between the ages of three and five, and both parent and child could participate in physical activity. We enrolled children in the upper end of normal weight and overweight body mass index percentiles (≥50th and <95th assessed by trained research staff), to reach those most at risk, but who were not yet obese. Full eligibility criteria and methods of the trial have been previously published [12]. The current analyses included caregiver-reported data from the baseline sample of 610 parent-child dyads randomized in the main trial, for which 2,126 families were approached, 1,607 met eligibility criteria, 839 gave informed consent, and 610 completed baseline data collection (Fig. 1).

### Procedures

Community liaisons (e.g., local pastors, directors of public daycare/pre-K programs, etc.) helped recruit participants from various community sites (e.g., daycares, doctors’ offices, pre-K programs, churches, community service programs, etc.) serving the target population. Trained bilingual research staff screened interested families for eligibility and conducted informed consent with those who were eligible. Informed consent and all study communication (including survey administration) were conducted in either English or Spanish according to participant preference [13]. At baseline, demographics and other self-reported measures were collected by guided verbal administration of a survey to address the low literacy rate of the population. Certified data collectors measured parents’ height and weight to calculate baseline body mass index (BMI).

### Measures

To assess parents’ perceptions of safety of their built environments, we developed a new instrument based on the Environmental Supports for Physical Activity Questionnaire [14]. The instrument consisted of 9 items that assess the walkability, bikeability, traffic safety, lighting, and crime safety of a participant’s neighborhood (Appendix). For this instrument, ‘neighborhood’ was defined as where the participant lives, or the area within one-half mile or a 10-minute walk from his or her home. Each item had three response options (disagree, don’t know/am not sure, agree), and was scored as follows: unsafe = 0, don't know/am not sure = 1, and safe = 2. Don’t know/am not sure was scored as an intermediate level of agreement to the safety or lack of safety of each item based on the idea that an uncertain perception of safety would be likely to impact use of the built environment and would have an intermediate effect when compared to either a perception that the environment was or was not safe.

A composite score was created as a sum of the 9 items, with higher scores representing safer perceived built environments. For those participants who were missing one of the nine items (*n* = 53, 8%), the participant’s own mean score on the remaining items was imputed for the missing item. Those who were missing values on more than 1 item (*n* = 5, <1%) were excluded from the analysis. The resultant scale had a range of 0–18 and demonstrated good internal reliability among items (Kuder-Richardson Formula 20 [KR-20] = 0.75). Additionally, a factor analysis indicated that the 9 items loaded onto a single factor.

To assess built environment availability, condition, and use for physical activity, a series of items was asked in a stepwise fashion using branching logic. Participants were first asked if they had each of the following six built environment structures available in their neighborhood (yes/no): public swimming pool, park or playground, sports field, walking track/trail, biking trail, and schools open after hours for public recreation. If yes, participants were asked about the condition of that structure as well as whether they used it. For example, a participant may have been asked the following series of items: 1) “Does your neighborhood have a walking trail?”; 2) [if yes] “In general, how would you rate the condition of the walking trail?; and 3) “Do you use your neighborhood’s walking trail?”. Responses to items assessing condition were scored on a 4-point Likert scale (Excellent (1), Good (2), Fair (3), and Poor (4)). Items assessing personal use were scored dichotomously (yes/no).

Three scores were derived from these procedures. The summary score for the *availability* of structures in the built environment was generated with a possible range of 0–6 structures. The *condition* of available built environment structures was the mean condition of all built environment structures a participant reported as available. The primary outcome variable, built environment *use* (i.e., the number of structures used), was a count of yes responses across all six structures (range 0–6).

### Statistical analyses

Demographics and baseline characteristics were summarized using means and standard deviations for continuous variables and proportions for categorical variables. Unadjusted associations were assessed using Spearman’s ρ or unadjusted logistic regression. To predict the dichotomous outcome of *any* use of the built environment, we fit a single logistic regression model including the availability, condition, and safety of the built environment. The model controlled for age, gender, ethnicity (Latino vs. non-Latino), marital status (single vs. living with a partner/married), employment status (any employment vs. unemployed), Supplemental Nutrition Assistance Program (SNAP) enrollment (yes vs. no), BMI (continuous kg/m^2^), and self-efficacy as measured by the 4-item perceived competence scale [15]. We initially included the interaction between condition and safety, to test the hypothesis that the effect of condition of the environment on use would differ according to safety level, with condition having a larger effect when safety was higher. However, the interaction term between perceived safety and condition was essentially zero and not significant. Consequently, we re-fit the models without the interaction term (which did not alter the other coefficients) and present the results from the more parsimonious models.

Then, to model the number of structures used (count data), we fit a Poisson regression model that included the condition and safety of available built environment structures, controlling for the same covariates as the logistic regression model. Poisson regression is the typical method used to analyze outcomes that are count data. However, it is susceptible to overestimating the significance of predictors when the outcome deviates from a Poisson distribution (i.e., overdispersion). We determined that Poisson regression was appropriate for the current analysis because the mean of the outcome was approximately equal to its variance (1.6 and 1.9, respectively), and the non-significant goodness-of-fit deviance statistic failed to reject the null hypothesis of good model fit (*p* = 0.6).

As a pre-specified secondary analysis to avoid obscuring significant findings from generating a composite outcome [16, 17], we also fit six additional logistic regression models to predict use of each of the six built environment structures as the outcome, using the same set of predictors and covariates described above.

All analyses were conducting using SPSS version 23.

## Results

Twenty-six percent of the participants in the baseline sample reported that none of the queried built environment structures existed in their neighborhood. Among the 452 participants who reported availability of at least one built environment structure, 53% reported fewer than three (M = 2.7, SD = 1.5). Of the participants who reported having at least one built environment structure available, 406 (90% of 452) had complete data on built environment use, condition, and safety thus constituting our analytic sample for subsequent analyses.

Demographic characteristics of the 406 individuals in the analytic sample are reported in Table 1. The average age of participants was 32 (SD 6.1) years and the average age of their children was 4.3 (SD 0.88) years. The majority of caregivers were women (98.5%) and self-identified as Hispanic/Latino (90.2%). Three-quarters of the sample was enrolled in SNAP, 39.5% were employed, and 17.8% were not married or living with a partner. The average caregiver BMI was 29.8 (SD 5.8) kg/m^2^.

**Table 1:** Baseline demographics and built environment features by use of the built environment

	Overall *N* = 406	Non-Users *N* = 65	Users [of any] *N* = 341
Demographic Characteristic			
Age (years), *mean (SD)*	32.0 (6.1)	32.1 (6.8)	32.0 (5.9)
Gender (female), *n(%)*	400 (98.5%)	63 (96.9%)	337 (98.8%)
Ethnicity (Latino), *n(%)*	366 (90.2%)	56 (86.2%)	310 (90.9%)
Marital Status (single), *n(%)*	72 (17.8%)	12 (18.8%)	60 (17.7%)
Employed, *n(%)*	160 (39.5%)	24 (36.9%)	136 (40.0%)
SNAP participant, *n(%)*	306 (75.6%)	50 (78.1%)	256 (75.1%)
BMI (kg/m2), *mean (SD)*	29.8 (5.8)	30.2 (5.2)	29.8 (5.9)
Self-Efficacy, *mean (SD)*	6.4 (3.0)	6.3 (2.9)	6.4 (3.1)
Built Environment Feature			
Number of structures available, *mean (SD)*	2.8 (1.5)	1.8 (1.0)	3.0 (1.5)
Average condition of available structures, *mean (SD)*	2.4 (0.8)	2.6 (0.9)	2.3 (0.7)
Perceived Safety of the Built Environment, *mean (SD)*	11.3 (5.0)	10.6 (5.2)	11.5 (5.0)

*SNAP* supplemental nutrition assistance program

Summary statistics for availability, condition, and use of each of the six built environment structures are described in Table 2 for the 406 participants in the final sample. Parks and playgrounds were most often reported as available (71.0%) and most often used when available (88.9%). Conversely, schools being open after hours for public recreation were infrequently reported as available (27.1%), but often used when available (52.4%). Overall, 83.9% reported using at least one structure. Average perceived condition was between good and fair for each of the six structures queried.

**Table 2:** Availability, use, and condition of built environment structures for physical activity

Built Environment Structure	Availability N (%)^a^	Use N (%)^b^	Condition Mean (SD)
Public Swimming Pool	162 (41.4%)	82 (51.3%)	2.3 (0.8)
Park or Playground	283 (71.0%)	247 (88.9%)	2.4 (0.9)
Sports Field	185 (47.2%)	89 (48.1%)	2.4 (0.8)
Walking Trail	190 (48.5%)	144 (76.2%)	2.1 (0.7)
Biking Trail	109 (28.7%)	53 (49.1%)	2.2 (0.7)
School open after hours for public recreation	65 (27.1%)	33 (52.4%)	2.0 (0.8)

^a^Availability is presented as the percent of valid responses for each item

^b^Percentage for use reflects the proportion of those who use the structure conditioned on the number who reported that structure as available

Using unadjusted logistic regression to test the bivariate relationships between availability, condition, and safety with any built environment use we found the following. The average number of structures available was twice as high for those who reported any use versus those who reported no use (unadjusted OR = 2.14, *p* < 0.001). The average condition of the built environment was 80% higher for those who reported using at least one structure versus those who reported no use (unadjusted OR = 1.8, *p* = 0.001). Perceived safety of built environment structures did not differ between those who reported using any structure and those who reported that they used no structures (unadjusted OR = 1.0, *p* = 0.2). Poorer condition of the built environment was correlated with a less safe built environment (ρ = 0.3, *p* < 0.001).

As shown in Table 3, results from the adjusted Poisson regression model demonstrated that the number of built environment structures used was positively associated with the total number of structures perceived as available and with the average condition of the built environment but not significantly associated with perceived safety, controlling for all else in the model. The adjusted logistic regression model provided similar findings, demonstrating that the perceived number of built environment structures in the neighborhood and the average condition of the built environment were associated with close to twice the odds of using the built environment for physical activity, controlling for all else in the model. Perceived safety was not significantly associated with use of the built environment. Finally, in individual adjusted logistic regression models for each structure measured, no significant associations were found between use of any of the individual built environment features and the perceived safety scale.

**Table 3:** Adjusted associations of built environment features with use of structures designed for physical activity

Built environment features	Number of structures used^a^			Use of any structures^b^		
	B	95% CI	P	AOR	95% CI	p
Number of structures available	0.34	0.31, 0.37	<0.001	2.34	1.73–3.15	<0.001
Average condition of available structures	0.19	0.12, 0.27	0.001	1.71	1.16–2.53	0.007
Perceived safety	0.00	−0.01, 0.01	0.7	0.97	0.91–1.03	0.3

Note: In two separate models (Poisson and Logistic) the same three main predictors were included: the number of structures available, the average condition of those structures, and the perceived safety of those structures, controlling for covariates. Both models controlled for the following: age, gender, ethnicity, marital status, employment status, Supplemental Nutrition Assistance Program (SNAP) enrollment, BMI, and self-efficacy

^a^Poisson regression

^b^Logistic regression

## Discussion

The built environment can be a powerful mechanism for facilitating physical activity. In this sample of primarily low-income, Latino parents, both perceived availability of structures and perceived condition of the built environment were associated with higher self-reported use of the built environment for physical activity. Specifically, the odds of using any structure in the built environment for physical activity were 2.3 times higher for every additional structure reported as available in the neighborhood. Similarly, for every one-unit improvement in perceived physical condition of the built environment, the odds of using the built environment increased by 1.7 times.

Recognizing the importance of parenting practices for childhood physical activity, previous literature has identified associations between parent perception of environmental attributes and both child overweight and use of the built environment [18–21]. Even when considering objective measures of built environment safety, parent perception of the built environment is most strongly associated with physical activity parenting practices [10]. The modest relationships reported in this analysis are consistent with previous literature and with the expected contribution of parent perception of built environment to built environment use [22]. This study adds to the literature by highlighting the contribution of parent perception of built environment condition as a primary driver of built environment use, even when controlling for other individual and social factors.

Participant report of features of the built environment may reflect actual availability and condition or it may represent a parent’s lack of knowledge of existing structures in their community. In particular, participants were not often aware that schools were open after hours for physical activity, but over half of participants who reported their availability used the schools for physical activity. This explanation is consistent with our previous work in this community, which found that Latino families did not think that local recreation centers were intended for their use, and that their use of local recreation centers increased once they had been taught how to engage those services [23]. In addition, 26% of participants reported that none of the queried structures existed in their built environment, which suggests that many low-income Latino and African American families may not have any of these structures in their neighborhood, may not know that they exist, and/or may not be utilizing existing neighborhood structures to support the health of their families. Marketing available resources as well as building and maintaining built environment structures in underserved communities warrants attention from local stakeholders.

In this sample, perception of built environment safety was not associated with built environment use for physical activity. Even though participants reported a high frequency of unsafe built environment characteristics, condition of the built environment, not safety, was the primary driver of reported use. Despite this lack of an observed association, this study advances the field by describing the use of a composite built environment safety score. In previous work, measures of built environment safety have relied on global assessments [17]. The scale presented in this study includes specific measures of built environment safety across a wide range of potential safety domains. The scale utilized in this study had high internal consistency and good construct validity (i.e., associated with condition of the built environment). We suggest that future studies use such a composite measure of built environment safety features to better characterize potential safety-related concerns in the built environment. Future studies should also include an indicator of safety-related features that are more relevant to parks or playgrounds in order to understand more fully the relation between safety concerns and built environment use.

The findings regarding the influence of number of structures available on *any* use of such structures aligns with previous research among Latinos showing that parents are more likely to engage with their children in physical activity when there are more places for physical activity in their neighborhoods [10]. These results suggest that city planning and urban design has the potential to enhance physical activity in populations disproportionately affected by the obesity epidemic, potentially reducing the public health burden [8].

This study had several limitations. Parent perception of built environment features (i.e., availability, condition, safety) is only one of many potential contributors to built environment use, thus, despite including a wide range of covariates, we may not have accounted for other important contributors to built environment use. Because we could only examine condition and use among those who reported a built environment structure, we eliminated those families with no structures, who might represent those most in need of intervention. Moreover, our measure of built environment use was general, and thus our analyses could not address determinants of frequency of use or whether children participated with their parents. Although urban, low-income Latino families’ physical activity is important to understand because of their heightened risk for obesity, our findings may not generalize to other parents in different geographic locales. This study considered only perceived features of the built environment and did not include directly observed measures. Nevertheless, this study advances our understanding of the importance of built environment structure availability and condition that can enhance the potential for physical activity in young Latino children. Finally, the safety composite instrument scores a response of “I don’t know” between Yes and No, which may have inaccurately categorized perception of safety. In addition, many of the items in the safety composite instrument are relevant to pedestrians, which may not impact participants’ use of queried structures if they use a vehicle to access them. For example, the most available structure for physical activity was a park or playground, reported by 71%; if the participants did not walk or ride bikes along sidewalks to access these playgrounds, the safety measure used in this study may not have captured the safety concerns relevant to playground structures (e.g., loitering adults, debris). Finally, 98.5% of participants in this study were female caregivers, which may limit the generalizability of findings to entire families, where fathers, or other caregivers in households with multiple adults, are responsible in some capacity for overseeing the child’s physical activity.

## Conclusions

Among urban, low-income, Latino and African American families with young children at risk for obesity, a significant percentage reported no available structures designed for physical activity in their neighborhoods. Among those with any such structures available, built environment use was influenced by parents’ perceptions of the structures’ condition as well as the number available. These findings call for efforts to improve and provide more publicly available structures for physical activity, particularly in neighborhoods with residents disproportionately affected by childhood obesity.

## Appendix

To assess parents’ perceptions of safety of their built environments, we developed a new instrument based on the Environmental Supports for Physical Activity Questionnaire [14]. The instrument consisted of 9 items that assess the walkability, bikeability, traffic safety, lighting, and crime safety of a participant’s neighborhood.

**Table 4:** Survey items

Built environment safety scale items:	Response options
1. There are sidewalks on most of the streets in my neighborhood	0: Disagree 1: I don’t know/am not sure 2: Agree
2. The sidewalks in my neighborhood are well maintained	0: Disagree 1: I don’t know/am not sure 2: Agree
3. Overall, my neighborhood is a safe place to walk	0: Disagree 1: I don’t know/am not sure 2: Agree
4. Overall, my neighborhood is a safe place to ride a bike	0: Disagree 1: I don’t know/am not sure 2: Agree
5. The traffic in my neighborhood makes it difficult to walk or ride a bike in my neighborhood	0: Agree 1: I don’t know/am not sure 2: Disagree
6. Many drivers exceed posted speed limits while driving in my neighborhood	0: Agree 1: I don’t know/am not sure 2: Disagree
7. My neighborhood streets are well lit at night	0: Disagree 1: I don’t know/am not sure 2: Agree
8. The crime rate in my neighborhood makes it unsafe to go on walks	0: Agree 1: I don’t know/am not sure 2: Disagree
9. Unattended dogs in my neighborhood make it unsafe to go on walks	0: Agree 1: I don’t know/am not sure 2: Disagree

Nine items comprise the built environment safety scale. Each item had three response options: I agree; I don’t know/am not sure; I disagree. Items were scored so that higher scores represented safer environments. Thus items 5, 6, 8, and 9 were reverse coded as shown above

## Abbreviations

AOR: Adjusted odds ratio
BMI: Body mass index
CI: Confidence interval
M: Mean
OR: Odds ratio
SD: Standard deviation
SNAP: Supplemental Nutrition Assistance Program
